# Editorial: Regulatory science: addressing uncertainties in medicines development, evaluation and use

**DOI:** 10.3389/fmed.2026.1835287

**Published:** 2026-04-16

**Authors:** Liese Barbier, Carla Torre, Rosanne Janssens

**Affiliations:** 1 European Medicines Agency, Amsterdam, Netherlands; 2 Faculdade de Farmácia, Universidade de Lisboa, Lisbon, Portugal; 3 Laboratory of Systems Integration Pharmacology, Clinical and Regulatory Science, Research Institute for Medicines of the University of Lisbon (iMED.ULisboa), Lisbon, Portugal; 4 KU Leuven, Leuven, Belgium

**Keywords:** academia, regulatory decision making, regulatory science, regulatory science research, uncertainty management

## Background

Improving the transparency of regulatory decision-making, including the management and communication of uncertainty, was highlighted as a key area of increased focus and importance in the Regulatory Science Strategy to 2025 of the European Medicines Agency (EMA) (1). This was further reinforced in the EMA Regulatory Science Research Needs 2021 and its 2025 update, which highlight the need and opportunity for research to advance how to identify, measure, evaluate, mitigate and communicate uncertainty across medicines development, regulatory decision-making and use (2, 3).

Uncertainty can be broadly defined as a lack of complete knowledge or understanding of a given situation. In medicines development and regulation, uncertainty arises primarily from limitations in available or lacking data and evolving scientific knowledge. Scientific knowledge is inherently incomplete and ever evolving, consequently, decision-makers must base their decisions on best available evidence while identifying, characterizing and weighing the uncertainties that remain. All medicinal products are associated with certain degrees of uncertainty throughout their life cycle, requiring appropriate ways of identification, management, communication and further mitigating.

In the context of regulatory decision-making, uncertainty may translate into difficulties in evaluating the quality, safety, efficacy, and overall benefit-risk balance of a medicinal product for a given patient population. Uncertainties in drug development and pre-authorization evidence generation also propagates downstream, affecting Health Technology Assessment (HTA), reimbursement decisions and clinical decision-making. Stakeholders across the medicine development ecosystem—including commercial and non-commercial developers, regulators, clinicians, academics, HTA bodies, patients and healthcare professionals—have a strong interest and benefit in improving how uncertainty is managed and communicated as to improve their decision-making processes and decision quality, allowing also for increased transparency toward the public, and in particular to patients (4).

However, currently, there is no comprehensive, generally agreed or structured approach for defining, categorizing, managing, and communicating uncertainties at various decision-making levels in drug development, evaluation, and use. Decisions affected by uncertainty span the full medicines life cycle, including the identification of unmet medical needs, prioritization and allocation of research resources, selection of clinical trial endpoints and outcomes, choice and validation of patient-reported outcome measures, regulatory benefit–risk assessment, safety and long-term safety, HTA and reimbursement decisions, and clinical treatment choices. Within regulatory practice specifically, practical tools to support uncertainty management and communication, such as templates, guidance documents, or checklists, remain limited for both assessors and developers. Key topics of uncertainty include the magnitude and durability of treatment effects, the appropriateness and clinical relevance of clinical trial endpoints, the interpretation of patient-reported outcomes, and the extrapolation of evidence to broader or underrepresented patient populations. These represent research foci receiving much attention from regulators, drug developers, scientists, clinicians and patients, where evidence-based insights and methodological advances would be highly valuable. For example, the EMA Regulatory Science Research Needs include the following uncertainty-related research needs (2, 3):

– Research on effective ways to communicate evidence-related uncertainties in European Public Assessment Reports (EPARs) to better support HTA decision-making.
– Studies to improve understanding of the potential effects of medicines on fetal and newborn health and in pregnant and breastfeeding women, thereby supporting more informed benefit–risk decision-making in these populations.
– Developing methods to quantify and report uncertainties in complex modeling and simulation models, such as diagnostic and reporting tools to facilitate regulatory decision based on these models.
– Developing standards and quality requirements for evidence generation methods to help address uncertainties, such as for the design, conduct, analysis, and reporting of patient-reported outcome (PRO) studies for regulatory submissions, and assessment of how such standards help evaluating the evidence from PRO studies during benefit-risk assessment and HTA.

This Research Topic aimed to stimulate addressing complex questions of identifying, managing and communicating uncertainty in medicines development, evaluation, and use. It also aimed to provide a spotlight for such research. This topic showcases regulatory science contributions that seek to advance conceptual, methodological and practical approaches and contribute views to uncertainty handling in practice.

Academic researchers, regulators and industry contributed to this Research Topic, with valuable cross-stakeholder collaborations as well. This reflects the multistakeholder nature of regulatory science research, wherein research is not only being advanced by academic researchers, but also by, and often in collaboration with, regulators, industry, patients and healthcare professionals (5). In total, this Research Topic comprises nine articles, resulting from the work of 59 authors. The Research Topic includes original research papers employing a combination of different quantitative, qualitative, and document analysis methods, as well as literature reviews and a policy brief.

We hope that this curated Research Topic will provide insight, inspiration and direction for researchers and stakeholders interested in advancing the topic of uncertainty identification, management and communication.

## Research contributions

### Uncertainty identification and management in regulatory decision-making

Several contributions focus on how uncertainties and mitigation strategies are discussed and applied within regulatory decision-making. Medicine regulators are tasked to determine whether a medicine's favorable effects outweigh its unfavorable effects based on the evidence submitted. The articles in this section examine how uncertainties surfaced in this process and propose approaches to improve their transparency, communication and systematic handling.

Zafiropoulos et al. introduce a new framework for describing and structuring uncertainties in benefit-risk assessment. The proposed model is structured around four core elements—Cause, Aspect, Type, Strategy (CATS)—and was developed based on issues identified in oncology marketing authorization applications and discussions with expert assessors, discussing the usefulness, understandability and relevance of the framework. While noting the need for further validation, the authors flag the potential usefulness of the proposed CATS structure to further enhance assessment report templates. For example, the assessment templates and resulting EPAR could include sections that require the assessor to describe the cause, aspect, type and strategy for each uncertainty identified.

In a second study, Hoek et al. conducted an ethnographic study of assessment procedures at the Dutch Medicines Evaluation Board (MEB). This work provides insights into how regulators evaluate applicants' claims regarding benefits and risks, and how uncertainties are actively sought and scrutinized. The findings illustrate the effort intensive and context-dependent nature of uncertainty identification and suggest that future research could further explore the social and collaborative dimensions of regulatory decision-making.

To enable early access to promising medicines for patients with unmet medical needs, e.g. those with rare or poorly treatable diseases, regulators have implemented pathways to expedite access, including EMA's Conditional Marketing Authorization (CMA). Medicine developers may be granted a CMA for medicines that address an unmet medical need on less comprehensive clinical data than normally required. It is important to note that such a CMA can only be granted when a positive benefit risk balance of the medicine has been concluded with the benefit of the medicine's availability to the patient outweighing the risk inherent in the fact that additional data are still required, and this in addition to other criteria that need to be met. Once a CMA has been granted, the marketing authorization holder must fulfill specific obligations and this within defined timelines to provide comprehensive data post-authorization (6). In a policy brief, Maksimova et al. examine ethical considerations associated with the application of CMA, including on how to define what constitutes an unmet medical need. The authors propose a research agenda to work on through multi-stakeholder engagement involving clinicians, patients and ethicists.

Regulatory agencies operate under a duty to serve public health, with their legitimacy influenced by public perceptions. Son developed and validated a survey instrument to assess and measure the multi-dimensional reputation of public agencies, focusing on South Korea's medicines regulatory agency. The analysis identified three main dimensions of reputation—performance, procedure, and technical competence—suggesting that reputation management should extend beyond technical expertise to include procedural transparency and demonstrable performance.

### Leveraging patient experience and real-world data to support uncertainty mitigation

Patient experience data (PED) and real-world data can complement traditional evidence generated from clinical trials and help address uncertainties in medicine development, adding to the totality of evidence in regulatory evaluations (7). Decisions where PED can be especially useful include decisions concerning unmet medical needs and clinical trial endpoint selection. PED encompass Patient-Reported Outcomes (PRO), Patient Preferences (PP) and any data that directly reflect the experience of a patient or a caregiver without the interpretation of another party. The EU regulatory approach to PED has been highlighted in a recent 2025 draft EMA Reflection Paper on Patient Experience Data (8).

Almeida et al., as part of a collaboration between European regulators and researchers, review the current landscape of PED in regulatory decision-making. The authors outline definitions and types of PED, describe potential use cases across the medicine's life cycle, and discuss methodological and digital challenges and opportunities. A lack of dedicated guidance is identified as a key barrier to more systematic inclusion of PED in regulatory submissions, alongside opportunities arising from digital tools that enable real-time collection of PED.

Additional contributions on this topic address patient-focused drug development through the analysis of patient perspectives shared on social media (Cimiano et al.). Authors explore how social media listening (SML) methods are increasingly applied and how evidence derived from social media can be used in the drug development process, for identification of unmet patient needs and its impact on regulatory decision making. The perspectives of patients, developers and regulators on the role of SML in drug development were reviewed, together with a review of current SML practices and use cases. Findings suggest that stakeholders are strongly aligned regarding the potential of SML for patient- focused drug development, while also identifying areas where regulatory guidance is needed to reduce uncertainty regarding the impact of SML as a source of PED. Also, in an EMA HMA expert review report the potential of social media data for regulatory use was investigated through an analysis of scientific literature. This work resulted in a set of regulatory use cases, identification of challenges and opportunities, and key points for consideration to guide future actions for establishing the value and enabling the use of social media in medicine regulation (9).

### Tool and landscape analysis contributions

The Research Topic also dedicates attention to methodological advances for predicting the submission frequency of periodic safety update reports (PSURs). Authors introduce and assess the EURD tool as data-driven application to support decision making on PSUR frequencies for a risk-based approach in the continuous evaluation of the benefit-risk balance in the post-authorization phase of a medicinal product (Gordillo-Maranon et al.).

Klein et al. contribute a European landscape analysis of approved synthetic polypeptides using biologics as reference medicinal products, calling for more transparency and global alignment in regulatory procedures for chemically synthesized products referencing biological originators.

### Advancing regulatory science for uncertainty management through collaboration

The contributions in this Research Topic illustrate how handling uncertainty is a shared challenge across disciplines, decision-making contexts and stakeholders. Progress in regulatory science solutions is driven not only by researchers but also by and in collaboration with regulators, industry, patients, and healthcare professionals. The articles in this Research Topic advance conceptual and methodological approaches to identifying, managing, and communicating uncertainty. The papers included in this topic illustrate how regulatory science can move beyond the implicit handling of uncertainty toward more explicit, structured, and transparent approaches. They highlight emerging tools, frameworks, and evidence sources that can support regulators and other decision-makers in navigating uncertainty more systematically, with the common goal of strengthening trust, transparency, and the quality of decision-making. Regulatory science research is key to system improvement and innovation and there are good reasons to enable, leverage and expect more in the future by bringing together all the actors involved.

## References

[1] EMA/HMA. European Medicines Agencies Network Strategy to 2025 - Protecting Public Health at a Time of Rapid Change (2020). Available online at: https://www.ema.europa.eu/en/documents/report/european-union-medicines-agencies-network-strategy-2025-protecting-public-health-time-rapid-change_en.pdf

[2] EMA. Regulatory Science Research Needs (version 1.0) (2021). Available online at: https://www.ema.europa.eu/en/documents/other/regulatory-science-research-needs_en.pdf

[3] EMA. Regulatory Science Research Needs - 2025 Update (2025). Available online at: https://www.ema.europa.eu/en/documents/other/regulatory-science-research-needs-2025-update_en.pdf

[4] Hogervorst MA Vreman R Heikkinen I Bagchi I Gutierrez-Ibarluzea I Ryll B . Uncertainty management in regulatory and health technology assessment decision-making on drugs: guidance of the HTAi-DIA Working Group. Int J Technol Assess Health Care. (2023) 39:e40. doi: 10.1017/S0266462323000375 37325997 PMC11570246

[5] Barbier L Moscariello P Leufkens HG Herold R Pasmooij M. A new European platform for advancing regulatory science research. Nat Rev Drug Discov. (2025) 24:485–6. doi: 10.1038/d41573-025-00024-y 39929997

[6] European Medicines Agency (EMA). Conditional Marketing Authorisation. Available online at: https://www.ema.europa.eu/en/human-regulatory-overview/marketing-authorisation/conditional-marketing-authorisation (Accessed March 12, 2026).

[7] Janssens R Barbier L Muller M Cleemput I Stoeckert I Whichello C . How can patient preferences be used and communicated in the regulatory evaluation of medicinal products? Findings and recommendations from IMI PREFER and call to action. Front Pharmacol. (2023) 14:1192770. doi: 10.3389/fphar.2023.119277 37663265 PMC10468983

[8] EMA. Reflection Paper on Patient Experience Data (2025). Available online at: https://www.ema.europa.eu/en/documents/scientific-guideline/reflection-paper-patient-experience-data_en.pdf

[9] EMA/HMA Social Media Data for Real World Evidence in Regulatory Decision Making. An expert review report for the HMA/EMA Big Data Steering Group. Draft report_Social Media Data for RWE in Regulatory Decision Making_BDSG Review (2024). Available online at: https://www.ema.europa.eu/en/documents/other/social-media-data-real-world-evidence-regulatory-decision-making_en.pdf

